# Perception of ADHD and Compliance in Relation to ADHD Medication

**DOI:** 10.1111/acps.70106

**Published:** 2026-05-18

**Authors:** Kim Berg Johannessen, Pelle Lau Ishøy, Tine Houmann, Eline Levin, Per Hove Thomsen

**Affiliations:** ^1^ Team for ADHD and Autism, Mental Health Centre Glostrup Mental Health Services in the Capital Region of Denmark Copenhagen Denmark; ^2^ Faculty of Health and Medical Sciences, Department of Clinical Medicine University of Copenhagen Copenhagen Denmark; ^3^ Child and Adolescent Mental Health Center Mental Health Services in the Capital Region of Denmark Copenhagen Denmark; ^4^ Child and Adolescent Mental Health Center Nordsjaelland Mental Health Services in the Capital Region of Denmark Copenhagen Denmark; ^5^ Department of Child and Adolescent Psychiatry, Research Unit Aarhus University Hospital Psychiatry Aarhus Denmark

**Keywords:** ADHD, compliance, gender, questionnaire study

## Abstract

**Introduction:**

ADHD medication has proven effective for treating ADHD in adults. Large registry‐based studies have generally reported high discontinuation rates over time and focused on different risk factors, including comorbidity, gender, and socioeconomic status. The present mixed‐model study examines self‐reported factors related to the discontinuation of ADHD medication in Danish adults. It specifically focuses on themes such as the perception of ADHD and the perceived beneficial and adverse effects of the medication, with the aim of identifying patients' reasons either for continuing or discontinuing the prescribed medication overall and in relation to gender.

**Objective:**

The present study explores patients' self‐reported reasons for continuing or discontinuing ADHD medication, as well as the factors that influence their decision to live with or without medication despite having ADHD.

**Methods:**

The present study employed a mixed‐model design and consisted of 1050 Danish adults who redeemed a prescription for ADHD medication for the first time between 2017 and 2019. Questionnaires were distributed by Statistics Denmark to 4748 adults, a representative sample of the 16,284 Danish adults who redeemed a prescription within that period. Discontinuation was defined as a gap of 12 months between redemptions, and questionnaires were sent to an equal number of patients who continued or discontinued ADHD medication. A series of 2 (continuation vs. discontinuation) × 2 (male vs. female) two‐way ANOVA was conducted for different main themes, all of which were derived from six patient interviews.

**Results:**

The patients who continued medical treatment were more likely to perceive ADHD as a biological illness, whereas those who discontinued treatment were more likely to perceive ADHD as an illness constructed by society. Furthermore, patients who continued medical treatment reported that the medication had a more positive influence on their lives, whereas those who discontinued the medication reported experiencing more negative feelings. Finally, males were more likely than females to perceive ADHD as a socially created illness, while females reported higher scores than males for taking ADHD medication for themselves, being able to work or attend school, being social, and being able to enjoy peaceful alone.

**Conclusions:**

The present findings suggest that the perception of ADHD as either a biological or a social construct is central to patients' decisions to continue or discontinue ADHD medication. Moreover, interesting gender differences were found regarding the perception of ADHD and the reasons for taking ADHD medication. From a clinical perspective, these findings underscore the importance of understanding the individual patient's perception of ADHD and highlight the need for relatively close clinical monitoring of patients with ADHD due to their high risk of discontinuing medication.

## Significant Outcomes and the Limitations

**Outcomes:**

- Patients who continued ADHD medication were more likely to perceive ADHD as a biological illness, indicating that perception influences treatment adherence.
- Gender differences were identified, with females showing higher compliance and reporting more personal reasons for taking medication, while males perceived ADHD as a socially constructed illness.
- The study highlights the need for personalized psychoeducation and support strategies for ADHD patients, particularly to address social stigma and perceived side effects

**Limitations:**

- The study is limited by potential selection bias, as non‐responders may have different compliance rates compared to those who participated.
- Discrepancies between self‐reported medication status and objective data may affect the validity of findings regarding medication continuation and discontinuation.

## 1 Introduction

ADHD is a widely recognized neurodevelopmental disorder and there is a strong scientific and clinical consensus that it constitutes a valid psychiatric diagnosis [1]. A comprehensive re‐analysis of data on ADHD incidence and prevalence across 204 countries from 1990 to 2019 indicates that the incidence of ADHD has increased over time [2], Furthermore, the use of medication to treat ADHD has increased significantly in recent decades ([3, 4]). Several studies have demonstrated that medication has a significant positive effect on what is understood to be the core symptoms of ADHD, including inattention, hyperactivity, and impulsivity [5]. Furthermore, studies on psychopharmacological treatment—hereafter referred to as medicinal treatment—in adults with ADHD suggest that medication to some degree also provides additional positive benefits, often perceived as related to the ADHD construct, such as emotional dysregulation [6] and sleep disturbances [7]. Despite the well‐documented positive effects of medical treatment of ADHD (e.g., [8]), many adults diagnosed with ADHD discontinue their prescribed ADHD medication ([9, 10]). Medication discontinuation can lead to serious consequences, including symptom recurrence and functional impairment, underscoring the importance of understanding this issue. For instance, research has shown that discontinuing ADHD medication can result in a resurgence of symptoms that negatively affect daily functioning and quality of life [11, 12]. Therefore, understanding the factors that contribute to medical continuation is crucial for improving treatment outcomes and ensuring that patients receive the full benefits of their prescribed therapies.

Previous research has identified several factors associated with discontinuation of ADHD medication, including comorbidity, sex differences, socioeconomic factors, and perceived treatment effectiveness. However, most studies rely on nationwide registry data and therefore provide limited insight into patients' own motivations, experiences, and subjective evaluations of ADHD medication. As a result, there is still a need for studies that directly address patient perspectives on continuation versus discontinuation.

The biomedical approach to ADHD has been criticized for overlooking cultural and contextual influences on how symptoms are expressed and pathologized [13, 14]. Despite extensive research, ADHD remains a contested diagnosis among experts and laypeople [15], contributing to stigma that portrays individuals with ADHD as immature or unreliable [16] and to negative self‐image among adults with ADHD [17].

Gender‐related patterns also appear in the literature. Females with ADHD tend to show greater impairment in areas such as social functioning and mood regulation, whereas males more often show deficits in working memory and academic performance [18]. Although medication appears equally effective across sexes [19], males discontinue treatment more frequently [20, 21, 22]. Lower education, lack of family history of ADHD, and lower perceived medication efficacy have also been linked to discontinuation. Socialization processes may further contribute: females are often conditioned to be more compliant and to internalize difficulties [23, 24, 25], which may help explain their generally higher adherence to ADHD medication.

The authors of the present article recently published a nationwide, registry‐based cohort study involving 23,926 Danish adults who redeemed a prescription for ADHD medication for the first time between 2010 and 2015 [20]. The Danish registry study found that females were more compliant than males, with a 5‐year overall drug survival rate of 29% in females compared to 23.5% in males. Moreover, comorbidities such as substance use disorders, schizophrenia spectrum disorders, and affective disorders were identified as risk factors for medication discontinuation, whereas eating disorders and intellectual disabilities were associated with continuation of ADHD medication. However, more detailed information from patients is needed than what large registry studies can provide. The aim of the present study was to gather patients' own personal statements, as registry‐based studies are unable to provide an in‐depth understanding of patients' motivation for continuing or discontinuing ADHD medication.

## 2 Aims

In line with the previous registry‐based study by Ishøy et al. [20], we examined a subsample of adults diagnosed with ADHD who participated in that study. Using a mixed‐methods design, we assessed their perceptions of ADHD, the perceived impact of the disorder, and their experiences with ADHD medication.

Based on prior literature, we formulated the following hypotheses:

1. The continuation group will report more positive effects of ADHD medication and show better adherence compared with the discontinuation group.
2. The continuation group will report lower levels of perceived stigma and stronger beliefs that ADHD is a biological disorder.
3. Females will exhibit higher medication adherence than males.
4. Females will report more internalizing difficulties related to ADHD, whereas males will report more externalizing difficulties.

## 3 Materials and Methods

### 3.1 Participants—Attrition

Based on the 16,284 Danish adults who redeemed a prescription for ADHD medication between 2017 and 2019, a sample of 4748 patients (27.4%) was drawn at random by the Survey Department at Statistics Denmark (DST Survey) in 2022. All patients were diagnosed in accordance with national guidelines, specifically by a specialist in psychiatry or in child and adolescent psychiatry. Subsequently, a questionnaire was sent to these 4748 patients, asking about the patients' own experiences with the medical treatment of ADHD. The present study is based on the 1050 questionnaires (22.1%) that were returned to DST. Both completed and partially completed questionnaires were included in the study, as only a few participants had omitted individual responses. To check for differences between the patients who returned the questionnaire and the general patient population from the large registry study by Ishøy et al. [20]—that is, those who redeemed a prescription for the first time between 2017 and 2019—analyses were conducted to assess whether the questionnaire sample was representative (Table A1).

### 3.2 Design of the Questionnaire

The present study was based on a questionnaire developed to explore possible differences between patients who continue and those who discontinue their ADHD medication. The final questionnaire items were derived from interviews with six participants recruited through a network of collaborative partners and conducted by an independent interviewer (four males and two females). All interviewed participants had been diagnosed with ADHD within the past 2 years, had received psychopharmacological treatment for ADHD, and had at some point either temporarily discontinued their psychopharmacological treatment or permanently discontinued their prescribed ADHD medication (so both “continuous” and “discontinuous” were represented). Thematic analysis was employed to analyze the semi‐structured interviews, which were subsequently transcribed for analysis. The transcripts were coded to identify themes within the data, and related codes were grouped to develop overarching themes that reflected both shared and unique participant experiences. The final questionnaire included an item on the number of times patients had tried different types of ADHD medication, as well as nine themes derived from the interviews: (1) Patient's own perception of ADHD, (2) Challenges related to ADHD, (3) How ADHD affects patients' lives, (4) The medical treatment of ADHD, (5) Reasons for not taking ADHD medication daily, (6) Reasons for taking ADHD medication in general, (7) Reactions from family and friends, (8) Information received from the prescribing physician, (9) General mood. All questions were rated on a continuous 5‐point scale. (See Appendix A for questionnaire).

The questionnaire was specifically developed for this study on the basis of the interviews and has not been validated in a larger population, which represents a limitation of the research. Additionally, the instrument includes only the identified themes as headings, without any further subscales.

### 3.3 Discontinuation Versus Continuation

A gap of 12 months between prescription redemptions was defined as discontinuation, consistent with our registry‐based study [20]. Discontinuation was defined as a binary (yes/no) variable. Although previous studies have used 180 days between prescriptions to define discontinuation (e.g., [26]) a 12‐month period was chosen in the present study because, in Denmark, patients may redeem medication lasting up to 180 days if they are prescribed two different doses to switch between according to their present needs or if they take breaks during treatment. Furthermore, by choosing a long interval between prescriptions as the criterion for discontinuation, we sought to avoid inclusion of false discontinuation. Continuation patients do not pause their medical treatment for 12 months or more.

Data from the Health Data Authority's National Prescription Register were used to define continuance. However, 136 (31.3%) of 435 patients in the discontinuance group reported currently taking ADHD medication and 96 (15.6%) of the 615 patients in the continuance group reported not currently taking ADHD medication. Despite the discrepancies between self‐reported data and objective data with regard to continuance and discontinuance, the present study relies on the objective data and the definition of continuance versus discontinuance applied in the large registry‐based study from which the participants in the current study were drawn [20].

### 3.4 Statistical Analysis

To examine the interaction between medication status and gender in patients who discontinued the prescribed ADHD medication and the patients who still continued the ADHD medication, a series of 2 (continuation vs. discontinuation) × 2 (male vs. female) two‐way ANOVA was conducted. Table A2 presents the means of all questionaries measures across the four conditions, along with main effects and interactions. In addition, supplementary two‐way ANOVAs were conducted to address medication switching (i.e., number of medication changes) in relation to compliance and gender.

## 4 Results

### 4.1 Continuation Versus Discontinuation

First, we focus on continuation versus discontinuation where several main effects and a few interactions were observed. Patients who continued the prescribed ADHD medication scored significantly higher than those who discontinued ADHD medication on perceiving ADHD as a biological illness (*M* = 3.91 vs. *M* = 3.57; $\eta_{p}^{2}=.02$), ADHD as a positive trait (*M* = 3.08 vs. *M* = 3.82; $\eta_{p}^{2}=.01$), ADHD as something beyond personal control (*M* = 3.81 vs. *M* = 3.52; $\eta_{p}^{2}=.02$), and ADHD as a source of difficulties with practical tasks (*M* = 4.01 vs. *M* = 3.84; $\eta_{p}^{2}=0.01$).

In relation to the patients' experiences with ADHD medication, the continuation group scored significantly higher on taking the medication as prescribed (*M* = 4.39 vs. *M* = 4.10; $\eta_{p}^{2}=0.02$), and most notably, on perceiving the ADHD medication as effective for their ADHD symptoms (*M* = 4.25 vs. *M* = 3.36; $\eta_{p}^{2}=0.14$). Furthermore, the continuation group reported being significantly better informed by their prescriber about how the ADHD medication would help them (*M* = 4.00 vs. *M* = 3.81; $\eta_{p}^{2}=0.01$) and about potential side effects (*M* = 3.86 vs. *M* = 3.62; $\eta_{p}^{2}=0.01$), compared with the discontinuation group. Moreover, the continuation group scored significantly higher on all reported measures regarding reasons for taking the ADHD medication, most notably taking their ADHD medication for themselves (*M* = 4.55 vs. *M* = 4.22; $\eta_{p}^{2}=0.04$). In contrast, the discontinuation group reported fewer positive aspects of their ADHD (*M* = 4.01 vs. *M* = 3.84; $\eta_{p}^{2}=0.01$) when taking ADHD medication. Furthermore, the discontinuation group reported a stronger preference for treating their ADHD symptoms though approaches other than medicine (*M* = 3.53 vs. *M* = 2.52; $\eta_{p}^{2}=0.12$), worrying more about long‐term side effects (*M* = 2.92 vs. *M* = 2.60; $\eta_{p}^{2}=0.01$), reported higher levels of shame associated with taking ADHD medication (*M* = 2.03 vs. *M* = 1.82; $\eta_{p}^{2}=0.01$) and finally reported instances of taking ADHD medication not prescribed to them (*M* = 1.31 vs. *M* = 1.21; $\eta_{p}^{2}=0.01$). Finally, the discontinuation group reported that their family and friends were more opposed to ADHD medication than did the continuation group, and vice versa (*M* = 2.05 vs. *M* = 1.59; $\eta_{p}^{2}=0.05$).

With regard to shorter or longer medical breaks, the continuation group reported doing so because of forgetfulness in taking their medicine (*M* = 2.71 vs. *M* = 2.50; $\eta_{p}^{2}=0.01$) and difficulties picking up their prescriptions from the pharmacy (M = 2.24 vs. M = 1.89; $\eta_{p}^{2}=0.03$). In contrast, the discontinuation group reported taking breaks from ADHD medication due to physical side effects (*M* = 3.11 vs. *M* = 2.39; $\eta_{p}^{2}=0.06$), increased negative emotions (*M* = 2.94 vs. *M* = 2.26; $\eta_{p}^{2}=0.06$), a reduction of positive sides of ADHD (*M* = 2.66 vs. *M* = 2.24; $\eta_{p}^{2}=0.02$), difficulties relaxing (*M* = 2.62 vs. *M* = 2.17; $\eta_{p}^{2}=0.03$), lack of effect of the ADHD medication (*M* = 3.36 vs. *M* = 4.25; $\eta_{p}^{2}=0.13$), and a sense that medication made life more difficult (*M* = 2.57 vs. *M* = 1.98; $\eta_{p}^{2}=0.06$). Interestingly, the discontinuation group also reported significantly more often that they did not to believe that they had ADHD (*M* = 1.51 vs. *M* = 1.29; $\eta_{p}^{2}=0.04$) and expressed a general feeling of sadness (*M* = 3.54 vs. *M* = 3.23; $\eta_{p}^{2}=0.01$), compared with the continuation group.

Significant interactions were found only for three variables: ADHD as a positive trait (*F =* 4.58, $\eta_{p}^{2}=0$.01); ADHD causing difficulties with practical tasks (*F =* 6.23, $\eta_{p}^{2}=0$.01); and perceiving family and friends as more positive toward ADHD medication (*F =* 4.58, $\eta_{p}^{2}=$ 0.01). In all cases, the difference between the continuation and discontinuation groups was greater for females than for males.

### 4.2 Male Versus Female

No significant difference was found in the distribution of males and females across the continuation and discontinuation groups. See Table A3. (χ^2^ (1) = 0.02, *p* = 0.9; *N* = 1050; Cramer's V = 0.00).

As shown in Table A2, differences between genders, noteworthy main effects were found in relation to perception of ADHD and medicine continuation. In the present study, males scored higher on perceiving ADHD as a socially created illness. Females scored higher on reporting that ADHD made it difficult to be alone, that it was uncontrollable and that it caused difficulties with practical tasks compared with males. Males reported perceiving ADHD as a problem for others and as the reason for their loss of contact with family and friends. At the same time, they scored higher both on perceiving ADHD as having a negative influence on their lives and as having no influence at all. Furthermore, gender differences were also found in reasons for taking ADHD medication. Females reported higher scores for taking ADHD medication for themselves, for being able to work or attend school, for being social, and for being peaceful when alone. In contrast, males scored higher on taking ADHD medication for the sake of their family compared with females. Lastly, compared with females, males reported a higher tendency to use ADHD medication not prescribed to them, to take medicine only when needed, and to question the ADHD diagnosis.

The two‐way ANOVA, with the mean number of medicine changes (range = 0 to 5 +) as the dependent variable and continuation (*M* = *0.93* SD = *1.24*) versus discontinuation (*M* = *1.06* SD *= 1.32*) × male (*M = 0.97* SD *= 1.29*) versus female (*M* = *0.98* SD = *1.26*) as independent variables, showed no significant main effects or interactions.

## 5 Discussion

We predicted a difference between genders in continuation status. However, no significant difference was found between males and females with regard to continuing or discontinuing ADHD medication. This finding contrasts with the general findings that males discontinue medical treatment more often than females [21].

Regarding patients' experiences with ADHD medication, the continuation group reported more positive effects on their ADHD symptoms, a result that has been reported in several previous studies (e.g., [10]). On the other hand, the discontinuation group reported an increase in negative emotions and a reduction in the positive sides of their ADHD when taking their medication, which are common reasons for discontinuation [27]. The discontinuation group also reported greater concern about long‐term side effects, which has previously been found to negatively influence medicine continuation [28]. This heightened concern regarding side effects may be associated with the fact that the discontinuation group reported being less informed about possible effects and side effects, as satisfaction with caregivers' information about medication has been shown to be associated with ADHD medication continuation [29]. Moreover, the present study found that the discontinuation group reported being feeling sad for no apparent reason. This may reflect factors unrelated to medication, such as emotional dysregulation associated with ADHD, or it may be linked to the poorer quality of life often reported when discontinuing ADHD medication [30]. Even though the continuation group reported better adherence to taking their ADHD medication as prescribed compared with the discontinuation group, the continuation group did not report full compliance to the prescribed medication. This may reflect unmet treatment needs in adults with ADHD throughout the day [31], leading patients to divide doses during the day to achieve that effect, take medical breaks, or use others' ADHD medication. These behaviors were reported in both groups, though they were more prevalent in the discontinuation group and particularly among men. The reported reasons for medication breaks are consistent with common reasons for medication discontinuation, such as lack of symptom control and dosing inconvenience [27].

It is well established that not all patients maintain perfect adherence, and classifications of adherence vary. Many of the same reasons for not taking ADHD medication in general were also cited as reasons for taking shorter or longer medication breaks. Whereas the discontinuation group reported taking medication breaks due to various negative mental and physical side effects of ADHD medication, the continuation group reported forgetfulness as the general reason for medication breaks. Both explanations have been found to be predictors of discontinuation [32]. Finally, lack of effect of ADHD medication was also found more frequently in the discontinuation group, particularly among men, in relation to medication breaks—both factors that are generally associated with discontinuation [33].

Social support was also found to be relevant for medication continuation [27]. The continuation group, particularly females, reported receiving more support from family and friends regarding ADHD medication, whereas the discontinuation group reported more negative attitudes toward ADHD medication from family and friends. Furthermore, feelings of shame about taking ADHD medication were also higher in the discontinuation group, indicating that self‐stigma influences discontinuation. These findings are consistent with work by Sobanski and colleagues [22], who found that a lack of family history of ADHD was associated with discontinuation. This may reflect both a greater understanding of ADHD and medication, as well as greater normalization of ADHD within families with a history of ADHD.

Self‐rated quality of life has also been associated with adherence to ADHD medication [34], and Sobanski et al. [22] found that reasons for taking ADHD medication included both self‐ and observer‐rated efficacy. These results correspond with the present findings, in which the continuation group reported higher scores for taking ADHD medication for themselves, for being peaceful when alone, and, at the same time, for the sake of their family. Furthermore, being able to attend school or work and being social were also rated higher as reasons for taking ADHD medication in the continuation group, thereby allowing them to live a more fulfilling life [33]. Interestingly, gender differences were also found in the reasons for taking ADHD medication. Females reported higher scores for taking ADHD medication for themselves, for being able to work or attend school, being social, and for being peaceful when alone, compared with males. In contrast, males reported taking ADHD medication for the sake of their family more often than females. A review by Faheem et al. [18] found that females are more impaired in social functioning, which may account for the present gender difference regarding being social. However, the review also found that males are more impaired in relation to education, where females also score higher than males in the present study. Moreover, females are generally more likely to exhibit internalizing symptoms and experience lower self‐esteem [35]. This underscores the importance of the ability to be alone for mental well‐being. The findings of the present study support this notion, as females with ADHD reported greater difficulties being alone.

Differences between the continuation group and the discontinuation group were also found in relation to patients' understanding of ADHD and its impact on their lives. The continuation group reported that their ADHD made daily practical tasks more challenging, a finding commonly associated with the difficulties experienced by individuals with ADHD [36]. Additionally, they perceived their ADHD as uncontrollable. This perception of an inability to manage behavior without medication has also been linked to issues of continuation, as demonstrated in a study by Khan and Aslani [37]. In the present study, ADHD was rated as more uncontrollable by females than by males. This may be explained by the tendency of females to internalize their symptoms [35] or by the fact that they often have experienced a variety of other mental difficulties prior to being diagnosed with ADHD [38]. Despite these challenges, the continuation group also reported perceiving ADHD as a more positive trait and as having a positive impact on themselves. Although this may appear somewhat contradictory, it could be understood in the sense that, because the compliance group perceived ADHD as more biological and uncontrollable, they may also perceive positive aspects of their ADHD when well medicated, as this enables them to be better regulated and to live a more fulfilling life.

The conceptualization of ADHD as a neurodevelopmental disorder is widely recognized [1]. Consistent with this view, the present study found that the continuation group was more likely to perceive ADHD as a biological illness compared with the discontinuation group. Medicine continuation in relation to beliefs that symptoms are caused by a chemical imbalance has also been found with regard to unipolar depression [39]. In contrast, the discontinuation group perceived ADHD more as a socially constructed illness. These two perspectives on ADHD complement each other, consistent with the latest expert consensus on ADHD published in 2021. According to this consensus, ADHD is believed to rarely develop on the basis of a single gene or a single environmental risk factor. Rather, most cases are thought to be explained by the combination of multiple genetic and environmental risk factors, each exerting only a small effect [1]. Although ADHD is most likely a combination of both genetics and environment, a central finding of the present study is that different perceptions of ADHD are clearly related to continuation and discontinuation.

Continuation of ADHD treatment may indicate more severe or heritable forms of the disorder, while discontinuation could suggest a lower degree of impairment or difficulty. This distinction is crucial in the context of continuation versus discontinuation of medication, as it implies that individual treatment responses are influenced by the underlying severity of ADHD. Studies have shown that patients with more severe symptoms are more likely to benefit from ongoing treatment, highlighting the importance of continuous medication for this population [11, 12]. Conversely, individuals who discontinue ADHD medication may experience less impairment, which could lead to a higher likelihood of discontinuing treatment.

## 6 Limitations

Although the results of the current study revealed interesting findings, some limitations must be acknowledged. First, non‐responders to surveys have been found to demonstrate poorer compliance than responders to surveys [40]. Additionally, individuals from low socioeconomic status backgrounds are also known to have lower adherence rates [41], and since the present study only consists of 38.9% males, the result may to some degree reflect selection bias in our sample. Second, the discrepancies between self‐reported data and objective data regarding continuance and discontinuance support the critique by Khan & Aslani [37] of self‐reported medicine continuation due to bias and low validity. Still, it also addresses the issue of using registry data as an objective measure since patients may have redeemed their prescription for ADHD medication without actually taking it.

Third, the present study did not address development of tolerance and integration of non‐pharmacological interventions as possible reasons for discontinuation. Tolerance may reduce the effectiveness of pharmacological treatments, leading patients to seek alternative methods for managing their symptoms [28]. Additionally, various non‐pharmacological strategies—such as behavioral therapies, mindfulness techniques, and lifestyle changes—have been shown to effectively alleviate ADHD symptoms and improve overall functioning [42]. Forth, as the design was cross‐sectional it did not allow for causal interpretations, but only correlational findings.

Finally, a notable limitation of this study is the lack of information available regarding the timing of diagnosis, diagnostic procedures, and severity of ADHD. If the sample encompasses individuals diagnosed in childhood or adolescence, it is essential to consider whether their experiences and characteristics are comparable to those diagnosed in early or late adulthood. Such variability may affect the generalizability of the findings and should be addressed in future research.

## 7 Conclusions

This questionnaire study contributes to the limited knowledge about the factors that drive continuation versus discontinuation of medication for ADHD. Patients who continued medication were more likely to perceive ADHD as a biological illness, whereas those who discontinued were more likely to perceive ADHD as a socially constructed condition. These findings highlight the importance of understanding individual patients' perceptions of ADHD, the need for psychoeducation and comprehensive information about medication, and the value of relatively close clinical monitoring—particularly for males with ADHD, who have a higher risk of discontinuing medication, but also for females with ADHD, as they may present with more subtle symptoms.

Ideally, future studies should adopt prospective designs and include comprehensive data on symptom severity, treatment context, and patient experiences. Such investigations are vital for enhancing our understanding of these issues and for informing clinical practice.

## Author Contributions

Kim Berg Johannessen wrote the manuscript and performed the statistical analyses. Per Hove Thomsen provided ongoing critical and constructive feedback. All authors contributed to study design and hypothesis generation and approved the final manuscript.

## Funding

The authors have nothing to report.

## Disclosure

Medice Nordic Denmark financed DLIMI. Medice Nordic Denmark did not have any influence on the study design, statistical analyses, interpretation of data nor any writing or publishing.

## Conflicts of Interest

The authors declare no conflicts of interest.

## Data Availability

In Denmark, questionnaire studies do not require approval from an ethics committee or informed consent. The participants in this study did not provide written consent for their data to be shared with other parties; therefore, the data are not publicly available. The authors received no funding for this work.

## Appendix A

Questionnaire consisting of 9 different themes. All statements were rated on a continuous 5‐point scale from strongly disagree (1) to strongly agree (5).

1. Patient's own perception of ADHD.
  ○ My ADHD is a biological illness within me.
  ○ My ADHD is a personality trait of mine.
  ○ My ADHD is an illness that is created by society.
  ○ My ADHD is a positive trait about me.
  ○ My ADHD is a problem for those with whom I socialize.
2. Challenges related to ADHD.
  ○ My ADHD makes it difficult being alone.
  ○ My ADHD makes it difficult to be with friends and/or family.
  ○ My ADHD makes it difficult to go to school and/or work.
3. How ADHD affects the patients' lives.
  ○ The fact that I have ADHD is not something I can control
  ○ The fact that I have ADHD has a negative impact on my life.
  ○ The fact that I have ADHD has a positive impact on my life.
  ○ The fact that I have ADHD means that I am at a disadvantage financially.
  ○ The fact that I have ADHD makes it difficult to do practical things in everyday life (e.g., cooking, doing homework, cleaning).
  ○ The fact that I have ADHD is the reason I have lost contact with close friends and/or family.
  ○ The fact that I have ADHD has no impact on my life.
4. The medical treatment of ADHD.
  ○ I always take my ADHD medication as directed by my doctor/psychiatrist
  ○ My ADHD medication negatively affects my emotions (e.g., I feel sad, lethargic, irritable, anxious)
  ○ My ADHD medication reduces the positive aspects of my ADHD/ADD.
  ○ My ADHD medication is working on my ADHD/ADD
  ○ I would prefer to treat my ADHD without the use of medication (e.g., with exercise, mindfulness, therapy)
  ○ I am concerned about long‐term side effects of taking ADHD medication.
  ○ Taking ADHD medication every day makes my life more difficult
  ○ I am ashamed to take ADHD medication.
  ○ I take medication for my ADHD that is not prescribed for me.
5. Reasons for not taking ADHD medication every day.
  ○ I don't take my ADHD medication as directed because I feel physically ill (e.g., headache, nausea, fatigue, palpitations, shaking hands).
  ○ I don't take my ADHD medication as directed because ADHD medication negatively affects my emotions (e.g., I feel sad, lethargic, irritable, anxious).
  ○ I don't take my ADHD medication as directed because I have trouble remembering to take my ADHD medication.
  ○ I don't take my ADHD medication as directed because I forget to pick up my ADHD medication at the pharmacy.
  ○ I don't take my ADHD medication as directed because I only take my ADHD/ADD medication as needed.
  ○ I don't take my ADHD medication as directed because my ADHD medication reduces the positive aspects of my ADHD.
  ○ I don't take my ADHD medication as directed because I think the medicine is too expensive.
  ○ I don't take my ADHD medication as directed because I have trouble relaxing when I take my ADHD medication.
  ○ I don't take my ADHD medication as directed because my ADHD medication is not working for my ADHD.
  ○ I don't take my ADHD medication as directed because taking ADHD medication every day makes my life more difficult.
  ○ I don't take my ADHD medication as directed because I don't think I have ADHD/ADD.
6. Reasons for taking ADHD medication in general
  ○ I take ADHD medication for my own sake
  ○ I take ADHD medication for the sake of my family.
  ○ I take ADHD medication to be able to attend to my work and/or my school.
  ○ I take ADHD medication to be socialize.
  ○ I take ADHD medication so that I can find peace when I am alone.
7. How family and friends have reacted to the ADHD diagnoses
  ○ My family and/or my friends have helped me plan my everyday life.
  ○ My family and/or my friends encourage me to take my ADHD medication.
  ○ My family thinks it's a bad idea for me to take my ADHD medication.
8. Information received from the prescribing doctor
  ○ The doctor has explained to me about my ADHD/ADD disorder.
  ○ The doctor has explained to me how my ADHD/ADD medication would help me.
  ○ The doctor has explained to me what side effects I could get from my ADHD/ADD medicine.
9. General mood
  ○ I am often sad without knowing why.
  ○ I have a positive outlook on where my life is going.

**TABLE A1:** Demographics.

	Background population		Questionnaires returned		Chi2‐test
	*N*	%	*N*	%	
Gender					
Female	7655	47.0	642	61.1	
Male	8629	53.0	408	38.9	< 0.001
Age (as of 01.01.2022)					
18–30 years	8322	51.1	517	49.2	
31–50 years	6453	39.6	400	38.1	
51+ years	1509	9.3	113	12.7	< 0.001
Medicine status					
Continuation	9939	61.0	615	58.6	
Discontinuation	6345	39.0	435	41.4	*p* = 0.113

**TABLE A2:** ADHD beliefs, experiences, and treatment perceptions by gender and medication status (continuation vs. discontinuation).

	Continuation				Discontinuation				Main effects				Interaction	
	Male		Female		Male		Female		Male versus Female		Contin. versus Discont.			
	*M*	SD	*M*	SD	*M*	SD	*M*	SD	*F*	$\eta_{p}^{2}$	*F*	$\eta_{p}^{2}$	*F*	$\eta_{p}^{2}$
*My ADHD is…*														
Biological illness	3.81	1.36	4.03	1.11	3.67	1.18	3.53	1.33	0.20	0.00	11.8	0.02***	3.79	0.01
Personality trait	3.82	1.08	3.63	1.16	3.69	1.24	3.62	1.13	2.33	0.00	0.74	0.00	0.58	0.00
Socially created illness	2.07	1.19	1.85	1.07	2.32	1.34	2.15	1.17	4.93	0.01*	10.37	0.01**	0.94	0.00
Positive trait	2.90	1.11	3.17	1.03	2.87	1.18	2.80	1.07	1.64	0.00	6.94	0.01*	4.58	0.01*
Problem for others	3.27	1.09	3.01	1.11	3.25	1.20	2.85	1.19	15.36	0.02***	1.18	0.00	0.74	0.00
*My ADHD troubles me…*														
Being alone	2.17	1.25	2.46	1.35	2.12	1.26	2.39	1.29	8.56	0.01**	0.37	0.00	0.21	0.00
Being with others	3.33	1.22	3.27	1.22	3.20	1.23	3.30	1.29	0.46	0.00	0.33	0.00	0.85	0.00
School and/or work	3.82	1.14	3.95	1.13	3.92	1.19	4.01	1.04	1.69	0.00	0.91	0.00	0.10	0.00
*To me ADHD is…*														
Uncontrollable	3.70	1.16	3.88	1.11	3.39	1.23	3.58	1.07	4.97	0.01*	12.63	0.02***	0.00	0.00
Negative impact on me	3.74	1.01	3.52	0.95	3.65	1.07	3.58	1.00	4.19	0.01*	0.07	0.00	1.05	0.00
Positive impact on me	2.86	1.12	3.06	0.89	2.71	1.11	2.74	1.02	2.64	0.00	9.83	0.01***	1.26	0.00
Poor economic status	3.27	1.34	3.35	1.30	3.22	1.39	3.13	1.29	0.00	0.00	2.01	0.00	0.89	0.00
Practical difficulty	3.81	1.17	4.20	0.96	3.83	1.20	3.82	1.22	5.76	0.01*	4.98	0.01*	6.23	0.01*
Loss of social relation	3.24	2.25	2.92	1.30	3.12	1.37	3.05	1.32	3.98	0.01*	0.00	0.00	1.58	0.00
No influence on my life	1.69	0.92	1.53	0.81	1.78	0.93	1.67	0.91	4.07	0.01*	3.10	0.00	0.13	0.00
*The medical treatment…*														
As prescribed	4.26	0.91	4.42	0.78	4.06	1.15	4.14	1.10	3.62	0.00	15.31	0.02***	0.41	0.00
Neg. emotional impact	2.62	1.31	2.45	1.29	3.23	1.37	3.30	1.42	0.33	0.00	69.33	0.06***	1.93	0.00
Reduces positive sides	2.68	1.17	2.52	1.22	2.87	1.24	3.06	1.29	0.04	0.00	20.04	0.02***	4.58	0.01*
Effect on ADHD	4.20	0.86	4.20	0.91	3.38	1.19	3.27	1.24	0.80	0.00	166.60	0.14***	0.71	0.00
Other ways of treatment	2.76	1.30	2.56	1.27	3.62	1.30	3.63	1.25	1.27	0.00	133.62	0.12***	1.53	0.00
Long‐term side effects	2.79	1.37	2.62	1.33	3.06	1.37	2.93	1.36	2.92	0.00	10.95	0.01***	0.07	0.00
Increases life difficulty	2.38	1.20	2.15	1.15	2.95	1.32	2.92	1.30	2.63	0.00	70.46	0.07***	1.60	0.00
Shame	1.91	1.05	1.84	1.13	2.13	1.16	1.93	1.16	3.35	0.00	4.80	0.01*	0.80	0.00
Using others' medication	1.31	0.68	1.16	0.53	1.49	0.95	1.23	0.66	20.99	0.02***	7.38	0.01**	1.66	0.00
*Medication pauses*														
Physically ill	2.40	1.34	2.53	1.46	3.10	1.42	3.36	1.56	3.76	0.00	59.27	0.06***	0.40	0.00
Neg. emotional impact	2.35	1.30	2.24	1.32	2.93	1.43	3.12	1.53	0.16	0.00	59.18	0.06***	2.60	0.00
Forgetfulness	2.60	1.36	2.81	1.47	2.44	0.132	2.30	1.28	0.17	0.00	12.85	0.01***	3.49	0.00
Lack of medication	2.29	1.28	2.29	1.38	2.00	1.11	1.73	1.00	2.70	0.00	26.94	0.03***	2.68	0.00
Medication when needed	2.60	1.36	2.40	1.44	2.68	1.38	2.26	1.38	10.50	0.01***	0.12	0.00	1.26	0.00
Reduce positive sides	2.37	1.21	2.23	1.24	2.74	1.30	2.71	1.36	0.98	0.00	23.85	0.03***	0.43	0.00
Cost of medication	2.98	1.50	3.13	1.52	3.08	1.43	3.14	1.41	1.14	0.00	0.34	0.00	0.23	0.00
Difficulty relaxing	2.27	1.18	2.17	1.21	2.65	1.27	2.76	1.42	0.00	0.00	31.67	0.03***	1.45	0.00
Lack of effect	1.93	1.01	1.89	1.07	2.81	1.36	2.92	1.41	0.18	0.00	138.05	0.13***	0.70	0.00
Make daily life difficult	2.17	1.19	2.03	1.15	2.81	1.35	2.64	1.41	3.23	0.00	53.78	0.06***	0.02	0.00
Question diagnoses	1.44	0.76	1.23	0.61	1.74	0.94	1.61	1.04	8.95	0.01**	36.41	0.04***	0.35	0.00
*Reasons for medication*														
Myself	4.51	0.76	4.58	0.74	4.10	1.03	4.30	1.01	6.04	0.01*	38.25	0.04***	1.17	0.00
Family	3.36	1.42	3.34	1.41	3.32	1.32	2.96	1.43	4.48	0.00*	5.29	0.01*	3.44	0.00
Work and/or school	3.95	1.18	4.33	0.93	3.74	1.26	4.05	1.08	23.69	0.02***	12.03	0.01***	0.25	0.00
Being social	3.36	1.27	3.67	1.30	2.98	1.27	3.13	1.39	7.30	0.01**	29.55	0.03***	0.81	0.00
Peaceful alone	3.36	1.35	3.49	1.36	3.10	1.40	3.33	1.37	3.99	0.00*	5.14	0.01*	0.31	0.00
*My family/friends…*														
Help planning	2.67	1.30	2.68	1.37	2.77	1.31	2.47	1.33	2.76	0.00	0.33	0.00	3.33	0.00
Support medication	2.89	1.36	3.01	1.36	2.70	1.22	2.46	1.26	0.42	0.00	17.62	0.02***	3.94	0.00*
Against medication	1.65	0.86	1.62	0.91	2.08	1.12	2.12	1.20	0.01	0.00	46.70	0.05***	0.26	0.00
*Practitioner educated…*														
About ADHD	3.86	1.14	3.85	1.16	3.95	0.96	3.68	1.14	3.56	0.00	0.39	0.00	3.13	0.00
Effects of medication	3.95	1.00	3.96	1.04	3.86	0.93	3.75	1.03	0.60	0.00	5.15	0.01*	0.35	0.00
Side effects of medication	3.72	1.15	3.87	1.13	3.66	1.05	3.55	1.12	0.04	0.00	6.60	0.01**	3.16	0.00
*General feeling…*														
Often sad for no reason	3.23	1.17	3.32	1.26	3.51	1.26	3.52	1.23	0.39	0.00	9.06	0.01**	0.30	0.00
Positive toward future	3.36	1.18	3.22	1.24	3.32	1.31	3.14	1.27	3.82	0.00	0.57	0.00	0.03	0.00

* *p* < 0.05.

** *p* < 0.01.

*** *p* < 0.00.

**TABLE A3:** Gender distribution by medication status. Gender distribution in relation to medicine status.

Gender	Medicine status	Total	% within gender
Male	Continuation	238	58.3%
	Discontinuation	170	41.7%
Female	Continuation	377	58.7%
	Discontinuation	265	41.3%
